# Estimating paediatric normative values for nerve studies using clustering techniques

**DOI:** 10.1016/j.cnp.2026.02.006

**Published:** 2026-02-28

**Authors:** G.K. Cooray, D. Motan, K. Howse, L. Nastasi, J. Deeb

**Affiliations:** a Karolinska Institutet, Stockholm, 171 77, Sweden; b Great Ormond Street Hospital, Great Ormond Street, London, WC1N 3JH, UK; c UCL Great Ormond Street Institute of Child Health, London, WC1N 1EH, UK; d Queen’s Hospital, Romford, Greater London, RM7 0AG, UK

**Keywords:** Paediatric electroneurography, Normal data, Clustering technique

## Abstract

**Objective:**

To estimate normative values from mixed clinical paediatric electroneurography data using an unsupervised clustering approach.

**Methods:**

Electroneurography studies from paediatric patients (2009–2024) were analysed for common motor and sensory nerves. Motor parameters included distal motor latency, CMAP amplitude, duration, area, and conduction velocity; sensory parameters included SNAP amplitude and conduction velocity. Data were grouped into age windows, and within each, t-distributed stochastic neighbour embedding (t-SNE) was applied to identify the normative distribution. The mean, 5th, and 95th centiles were derived and modelled using exponential fits.

**Results:**

Normative values were estimated for ages 0–18 years. Motor amplitudes increased with age, and conduction velocities rose rapidly until 3–4 years before plateauing. Distal motor latency showed a brief early dip followed by an increase. Sensory amplitudes peaked between 1 and 8 years, while sensory conduction velocities increased sharply in the first year, then gradually declined.

**Conclusion:**

Unsupervised clustering can derive normative paediatric electroneurography values from heterogeneous clinical data, yielding trends consistent with published references.

**Significance:**

This data-driven approach is practical, generalisable, and enables identification of likely healthy individuals using multivariate electrophysiological parameters.

## Highlights

• Age related changes in nerve and muscle parameters show early onset dynamics.
• Clustering enables extraction of normative nerve study values from mixed populations.
• A pipeline is described for extracting a normative subpopulation from clinical data.

## 1 Introduction

The central and peripheral nervous systems continue to mature after birth. This maturation occurs rapidly in early life and gradually slows during adolescence (Ferriere et al., 1985, Aherne et al., 1971). Neurophysiological parameters and waveform characteristics reflect this process, showing rapid changes in infancy and early childhood, followed by stabilisation towards adult levels by adolescence (Garcia et al., 2000). Several tissues contributing to neurophysiological signals also undergo postnatal maturation, including muscles, neuromuscular junctions, and peripheral nerves. For example, muscles transition from polyneuronal to mononeuronal innervation (Gramsbergen et al., 1997). Muscle fibres increase in both diameter and length, and the endplate region enlarges proportionally (Slater, 2008, Kozák et al., 2025). These developments lead to higher action potential amplitudes recorded over the muscle and greater dispersion of muscle fibre activation due to the expanded endplate zones (Stålberg and Karlsson, 2001). Consequently, motor nerve conduction studies often show higher increases in both amplitude and duration of the compound muscle action potential (CMAP) during early development. Peripheral nerves undergo parallel structural changes. Axonal diameters increase until around age five, while myelin thickness and internodal distances (ranging from approximately 0.5 to 1.5 mm) continue to grow over a longer period (Schröder et al., 1988, Ferriere et al., 1985). These changes result in higher sensory nerve action potential (SNAP) amplitudes and significantly faster conduction velocities (Frenzel et al., 2024).

Electrodiagnostic testing (EDX), including electroneurography and electromyography (EMG), relies on comparing a patient’s results to age-matched normative data to assess the likelihood of peripheral nervous system dysfunction. However, establishing normative data for electrodiagnostic tests is inherently challenging (Pitt, 2012, Pitt and Kang, 2015). Healthy individuals have little incentive to undergo testing, which can be uncomfortable or painful. Consequently, available normative data for the paediatric population are limited and often based on older studies—particularly for EMG data in children and infants. In some instances, researchers have turned to clinical data to approximate normative values. This approach, however, presents two main challenges:

1. All patients undergoing testing typically have a suspected or confirmed clinical issue.
2. The dataset is likely to include values from individuals with active pathophysiological processes affecting the peripheral nerves or muscles.

Regarding the first challenge, although patients in clinical settings typically present with a phenotype or suspected disorder, not all recorded measurements fall outside the expected normative range. Moreover, many individuals exhibit localised pathology while retaining normal function elsewhere. These unaffected regions can still produce data that closely resemble those of a healthy population. To address the second issue, some studies have analysed the distribution of test parameters to estimate proxies for physiological (normal) values. The *e-norms* and *extrapolated reference values* methodology are two approaches; using data near the mode of a distribution to estimate a Gaussian distribution (or similar distribution), which can approximate normative values. These estimates have generally aligned well with previously published normative ranges, suggesting they offer reasonable proxies for healthy population data (Jabre et al., 2020, Jabre et al., 2015, Nandedkar et al., 2018, Nandedkar et al., 2021, Dunker et al., 2024).

In this study, we present a relatively straightforward method to assess the distribution of neurophysiological parameters using clinical data. Each motor and sensory nerve test yields multiple measured values, forming a multi-dimensional feature space. We transform the data into a two dimensional data space using embedding techniques, allowing for the identification of data clusters. Standard statistical methods are then used to define clusters that serve as proxies for normative populations.

## 2 Data and methods

### 2.1 Data

The data for this study were retrospectively extracted from neurophysiological investigations conducted at the Department of Clinical Neurophysiology, Great Ormond Street Hospital for Children, London, UK, between 2009 and 2024. Motor and sensory nerve studies were done according to laboratory specific protocols, please see the supplementary data. Recordings were drawn from routinely performed nerve conduction studies using Natus Dantec™ Keypoint® Systems (Natus Medical Inc., Middleton, USA). Motor nerve data included the median, ulnar, tibial, and peroneal nerves. Sensory nerve data were collected from the median, medial plantar, superficial peroneal, and sural nerves.

For motor nerve responses, the following parameters were extracted: compound muscle action potential (CMAP) amplitude, negative peak duration, area, onset latency, and conduction velocity. For sensory nerves, amplitude and conduction velocity were recorded. For sensory nerves, we recorded the sensory nerve action potential (SNAP) and calculated the conduction velocity. Orthodromic conduction velocity was measured for the median and medial plantar nerves, whereas antidromic conduction velocity was assessed for the sural and superficial peroneal nerves.

The total number of nerve conduction studies included in the analysis was as follows:

• **Motor nerves**:
  – Median (abductor pollicis brevis, APB): N = 1303
  – Ulnar (adductor digiti minimi): N=969
  – Tibial (adductor hallucis, AH): N = 1769
  – Peroneal (extensor brevis, EB): N = 2706
• **Sensory nerves**:
  – Median (mid-palm): N = 2715
  – Medial plantar: N = 3930
  – Superficial peroneal: N = 2901
  – Sural: N = 2047

Motor and sensory nerves not listed above, as well as recordings from alternative anatomical sites (e.g., digits II–IV for median sensory nerves), were excluded due to insufficient sample sizes, which precluded reliable estimation of normative distributions. Furthermore, F-wave responses were not routinely done for the motor nerve and were not included in this study.

In cases where bilateral measurements were available for the same nerve in an individual, only data from the left side were included. Within-subject averaging was avoided to prevent bias in the estimation of probability distributions.

### 2.2 Data clustering with t-SNE

Data for each motor and sensory nerve were analysed separately. For each nerve, data samples were ordered by age, and sliding windows containing 50 data points were constructed. The data sample from each window was then used for the clustering analysis. The normative cluster was identified, and its age was defined as the mean age of the subjects within that window. Please see supplement Data samples for each nerve were analysed following preprocessing, which included the removal of zero values. Motor nerve responses were characterised using five parameters: baseline-to-negative peak amplitude, duration of the initial negative peak, area of the initial negative peak, onset latency, and conduction velocity. These values were derived from compound muscle action potentials (CMAPs) recorded during distal stimulation, with conduction velocity additionally estimated from proximal stimulation. Each of the five parameters was investigated for all included motor nerves. Sensory nerve data were analysed using two parameters: sensory nerve action potential (SNAP) amplitude and conduction velocity. These were processed using the same approach as the motor nerves.

The resulting dataset was resampled and subjected to dimensionality reduction using t-distributed stochastic neighbour embedding (t-SNE), which projected the five-dimensional data into two dimensions for visualisation (Van der Maaten and Hinton, 2008, Linderman and Steinerberger, 2017).

Dimensionality reduction was performed using t-SNE as implemented in scikit-learn (version 1.6.1). The embedding was computed in two dimensions (n_components = 2) with a fixed random seed (random_state = 42) to ensure reproducibility. Perplexity was set adaptively as the minimum of 30 or one less than the number of available features (perplexity = min(30, len(features) - 1)), thereby accommodating datasets with limited sample size. The embedding was initialised with a random configuration (init = ‘random’) and optimised with a learning rate of 200.0 (learning_rate = 200.0).

All other hyperparameters were retained at their default values, including the use of the Barnes–Hut approximation (method = ’barnes _hut’), a maximum of 1000 optimisation iterations (n_iter = 1000), an early exaggeration factor of 12.0 (early_exaggeration = 12.0), and an optimisation tolerance of $1\times 10^{-7}$ (tol = 1e-7).

The resulting two-dimensional point clouds were then partitioned using K-means clustering (Arthur and Vassilvitskii, 2006). K-means clustering was conducted using the scikit-learn implementation (version 1.6.1). We tested 2–5 clusters and to mitigate sensitivity to initial centroid placement, the algorithm was repeated with ten random initialisations (n_init=10), and the solution with the lowest within-cluster sum of squares was retained. A deterministic random seed was applied (random_state=42) to ensure reproducibility of results. All remaining hyperparameters were kept at their default values: cluster centres were initialised using the k-means++ method (init=’k-means++’), the maximum number of iterations per run was 300 (max_iter=300), convergence was defined by a relative tolerance of 1 × 10^−4^, and the Lloyd algorithm was employed for optimisation (algorithm=’lloyd’). The optimal number of clusters was determined using silhouette analysis (Rousseeuw, 1987). Most nerve embeddings showed optimal clustering with two clusters; a few showed three. For consistency across analyses, two clusters were used for each nerve.

Clusters were subsequently labelled as “normal” or “abnormal” based on mean nerve/muscle amplitude, with the cluster exhibiting the higher mean amplitude defined as normal.

### 2.3 Continuous estimate of the normative distribution

As previously described, age was included as an independent variable in our model by estimating age-dependent normative distributions via a windowing strategy that progressed with increasing age. For clustering with t-SNE, we employed non-overlapping windows containing 50 data points each. For each window, we calculated the mean as well as the 5th and 95th percentiles to describe the distribution of each parameter. This approach mirrors how nerve conduction values are typically reported in clinical practise and has been used previously in the literature (Jabre et al., 2015, Jabre et al., 2020). Developmental trajectories were then modelled using a two-exponential function of the following form:

(1) $$f\left(\text{age}\right)=A-\left|B\right|e^{-\left|C\right|\cdot \text{age}}+\left|D\right|e^{-\left|E\right|\cdot \text{age}},$$

where A represents the asymptotic value, and B,C,D,E determine the amplitude and rate of exponential changes. For motor nerves, the second exponential term was omitted. The analysis was re-run using both exponential terms for modelling the initial changes in distal motor latency of the tibial nerve, as well as for all sensory nerve parameters. For each parameter (mean, 5th percentile, 95th percentile), scatter plots of raw data were generated. Curve fitting was performed using non-linear least squares (SciPy curve_fit) with heuristic initial parameter estimates.

### 2.4 Statistical test and coding

All statistical tests and coding in this study were performed in python 3.11.5. The code and data is available at https://github.com/gercoo/Normative_Data_Clustering. The statistical and mathematical methods used in the study were carefully reviewed by one of the co-authors for correctness and relevance (GC).

## 3 Results

### 3.1 Normative distribution — motor nerves

**Fig. 1:**
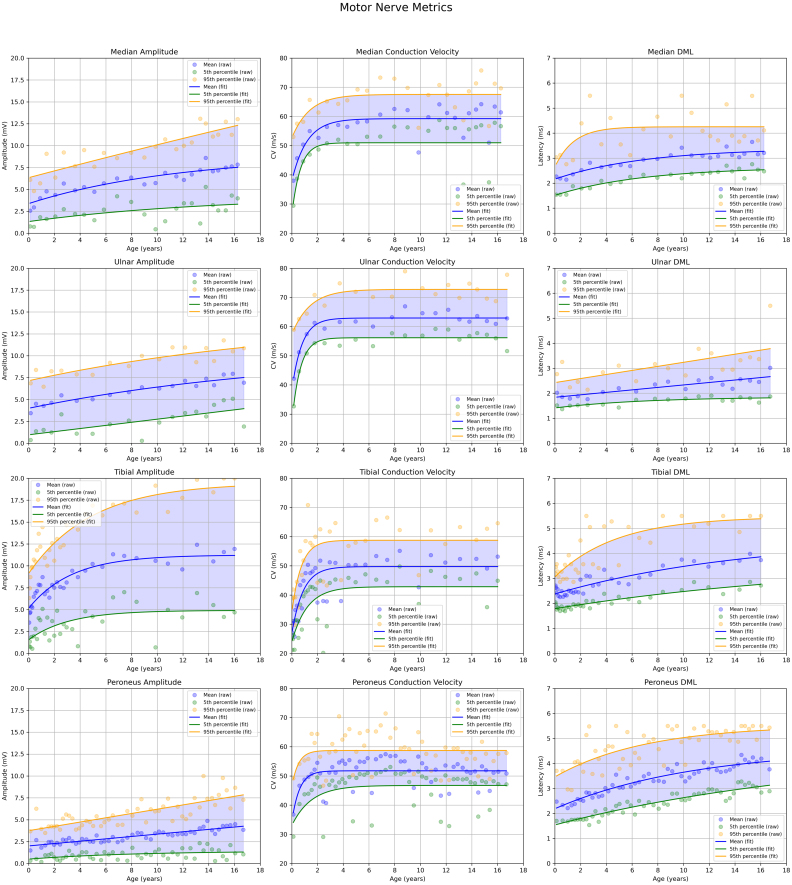
Normative graphs for motor nerves with amplitude, conduction velocity and distal motor latency. Different nerves were more or less frequently evaluated, as can be seen by the variation of the density of the data points. The increased sampling of certain nerves during the first two years of life enables a more granular view of the rapid changes seen during early development. On comparison of the conduction velocity with the amplitude and DML, there is a quick maturation of the CV parameters, while the other two show a more gradual increase.

**Fig. 2:**
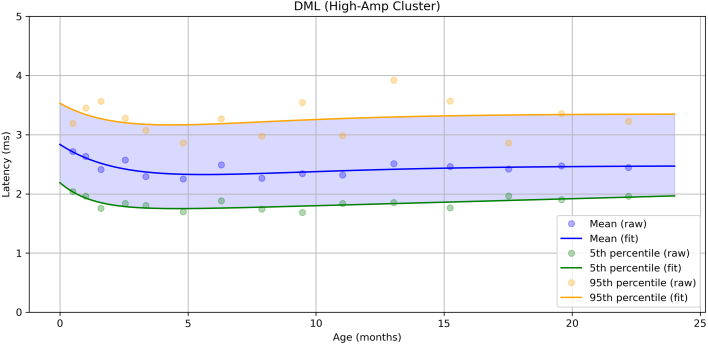
Normative tibial distal motor latency (DML) during the first two years of life. A rapid decline is observed over the first five months, followed by a gradual increase thereafter.

Across all motor nerves, CMAP amplitude demonstrated a gradual age-related increase over the 0–18-year period, with the rate of growth slowing with age (Fig. 1). The tibial nerve showed the greatest overall increase and the widest variability (difference between the 5th and 95th percentiles), whereas the other three motor nerves exhibited narrower ranges. Conduction velocity (CV) followed a similar developmental trajectory, with a rapid increase during the first years of life followed by a plateau. Variability also increased with age, as reflected by the widening gap between the 5th and 95th percentiles. Distal motor latency (DML) likewise showed age-related changes. When the data were modelled using a single exponential function, a relatively rapid increase in latency values was observed. However, closer inspection of the scatter plot near birth suggested the presence of an initial reduction followed by an increase. To test this, the tibial nerve data were refitted with two exponential components between 0 and 24 months, revealing a U-shaped curve (Fig. 2). Applying the same approach to the other motor nerves showed similar, although less pronounced, patterns. Sampling strategies differed between nerves: the tibial nerves were more densely sampled at younger ages, whereas the median, ulnar and peroneal nerves were sampled more evenly across ages.

### 3.2 Normative distribution—Sensory nerves

**Fig. 3:**
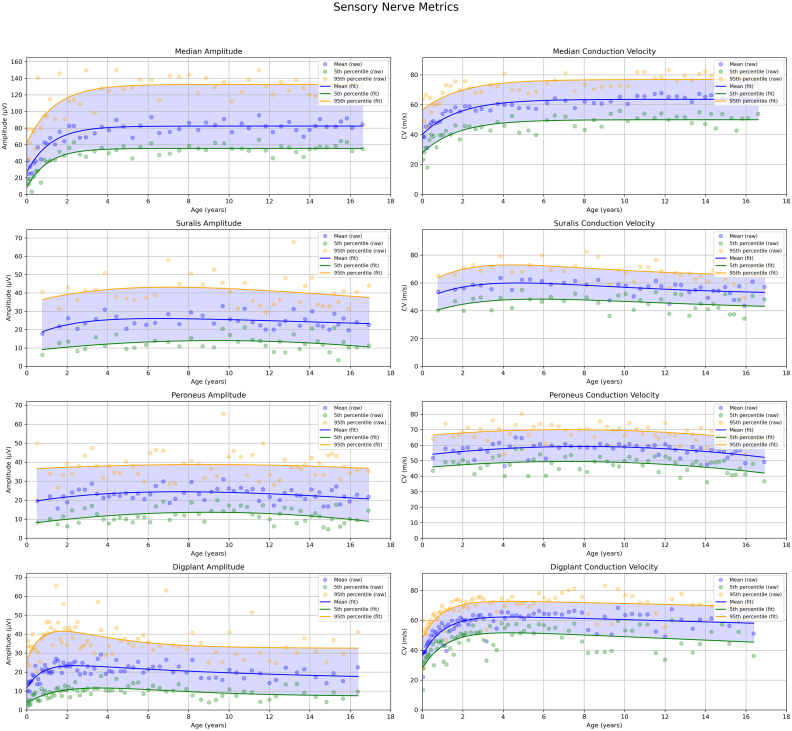
Normative graphs for sensory nerves with amplitude and conduction velocity. Different nerves were more or less frequently evaluated as can be seen by the variation of the density of the data points. The increased sampling of certain nerves during the first two years of life enables a more granular view of the rapid changes seen during early development. Both conduction velocity and amplitude show a rapid maturation during the first years after birth and then saturate.

The normative distribution of sensory nerves demonstrated clear age-related changes (Fig. 3). SNAP amplitudes showed a relatively rapid increase in the median and digital plantar nerves, followed by a gradual small decline with age. The sural and peroneal nerves exhibited a similar inverted U-shaped pattern, although the initial increase in amplitude was less pronounced and occurred later compared with the median and digital plantar nerves. Conduction velocity showed a comparable trajectory, most evident in the median and digital plantar nerves. The peroneal and sural nerves also displayed an inverted U-shape, but the changes were less marked than in the other two nerves. Sampling strategies differed between nerves: the median and digital plantar nerves were more densely sampled at younger ages, whereas the peroneal and sural nerves were sampled more evenly across ages but less frequently during the first months of life.

## 4 Discussion

This study investigated a large database of electroneurography recordings collected at a specialist paediatric hospital. Using unsupervised clustering techniques, the normative population for each nerve parameter was estimated as a function of age.

T-SNE was used for unsupervised clustering which projects high-dimensional data onto a two- or three-dimensional surface or volume, preserving the intrinsic statistical relationships between data points. As a non-linear technique, it adjusts distances regionally to maintain local structures within the data. The clustering is optimised by minimising the Kullback–Leibler (KL) divergence between the original high-dimensional dataset and its lower-dimensional representation (in this case, a two-dimensional surface). A key parameter in the algorithm is the number of neighbours (perplexity) used to define local clusters. This value depends on the dataset size and the nature of the underlying cluster structure; perplexity values between 20 and 50 typically yield robust results. Optimisation of the KL divergence is performed via gradient descent, which typically stabilises after around 5000 iterations. One strength of t-SNE is its data-driven approach to clustering, relying solely on the statistical properties of the dataset, making it relatively unbiased. In contrast to linear dimensionality reduction techniques such as PCA, t-SNE can capture complex, non-linear relationships between parameters (Van der Maaten and Hinton, 2008, Wattenberg et al., 2016, Linderman and Steinerberger, 2017). Additionally, the clusters formed by t-SNE do not assume Gaussian distributions. This is particularly important when analysing biological data, which often deviate from Gaussianity (Limpert et al., 2001) . Many conventional statistical methods require data transformations to enforce symmetry or normality, which t-SNE does not. However, because the clustering is unsupervised and unbiased, interpreting the meaning of each cluster requires careful post hoc analysis. In this study, a clinical perspective was used to guide interpretation, specifically focusing on the effects of disease processes such as conduction slowing or amplitude reduction. These effects informed the identification of clusters likely to represent normative versus pathological data. Typically, two to three clusters emerged for each neurography parameter, with two clusters being most common.

The estimated neurography values demonstrated an age-dependent dynamic. Parameters reflecting predominantly neuronal characteristics matured rapidly from birth, whereas those influenced by both neuronal and muscle fibre factors exhibited slower maturation. Conduction velocity, which depends almost exclusively on neuronal properties, showed clear evidence of rapid maturation in nerves with sufficient sampling soon after birth (Median, Tibial, and Digital plantar nerves). Similar patterns have been reported in the literature, where conduction velocity demonstrates relatively fast changes during the first year of life (Ryan et al., 2019, Lori et al., 2018, Rabben, 1995, Frenzel et al., 2024). In some nerves, conduction velocity appeared to decrease slightly after reaching a maximum, most clearly for the digital plantar nerve. This effect may reflect variability in the accuracy of statistical estimates at different ages. With increasing uncertainty in the mean at later ages, some apparent dynamics–particularly subtle changes—may not be statistically significant. Estimating parameter uncertainty more rigorously—for instance, through a Bayesian model to derive credible intervals (mean, 5th, and 95th centiles)–could help address this issue, although this would require substantial changes to the modelling pipeline. For example, inspection of the data indicates higher conduction velocities in the upper extremity compared with the lower, and noticeable differences between individual upper-limb nerves (e.g., median versus ulnar). Because the ulnar nerve was sampled far less frequently than the median nerve, the corresponding estimates are less reliable. A Bayesian fitting framework could have provided more robust uncertainty quantification in such cases, particularly where sampling density differs markedly between nerves.

Several nerves, such as the sensory Sural and Peroneal, did not show the sharp early increase in conduction velocity, most likely due to limited sampling in the first year of life. Their gradual decline in velocity with age may likewise lack statistical significance. In contrast, motor nerves displayed clearer age-related changes in conduction velocity, likely due to greater certainty in the estimated values. This difference may arise from methodological factors, as motor conduction velocity is typically measured over a much longer nerve segment than sensory conduction velocity. Another parameter independent of muscle physiology is the sensory nerve action potential. Its dynamics were somewhat more complex than those of conduction velocity. For the digital plantar sensory nerves, a rapid increase in amplitude was observed with a peak between 1 and 2 years of age, followed by a gradual decline and eventual plateau. A similar but less pronounced pattern was seen in the median sensory nerve, without the slow decrement at older ages. These digital plantar dynamics have been repeatedly reported in the literature; see (Kang, 2017) for a review. The observed changes likely result from a combination of axonal maturation (increase in axon diameter and progressive myelin compactification) and age-related increases in limb tissue mass. The sensory Sural and Peroneal nerves again did not display this early increase, most probably because of limited sampling during the first year. In contrast, parameters involving the compound muscle action potential (CMAP) showed slower maturation, with amplitudes continuing to rise into late adolescence. This overall trend is consistent with previous studies (Cai and Zhang, 1997, Ryan et al., 2019). While the difference in developmental dynamics between sensory and motor parameters may appear surprising, it aligns with underlying physiology: muscle development, including fibre diameter growth and expansion of the neuromuscular endplate zone, continues into adolescence, contributing to the prolonged rise in CMAP amplitude (Aherne et al., 1971, Lin et al., 1994). By comparison, peripheral nerve maturation—particularly axonal diameter increases—occurs earlier in life (Parano et al., 1993, Lang et al., 1985, Schröder et al., 1988, Ferriere et al., 1985).

Analysis of DML revealed a U-shaped trajectory: an initial decrease in values followed by an increase. This has been attributed to differing maturational rates of nerve, muscle, and limb length, and has been previously reported (Kang, 2017). In our data, this pattern could only be estimated with statistical significance for the Tibial motor nerve, again reflecting the need for frequent sampling during the first year of life.

The rapid maturation of nerve study parameters was evident in the results of this study. Capturing this pattern required a substantial dataset with dense sampling during the early years of life. This trend is illustrated in the figures, which show a continuous increase in normative values throughout infancy. The use of adult normative values in paediatric assessments may be acceptable for children of school age. However, their applicability diminishes rapidly with decreasing age and becomes particularly unreliable in infants and newborns due to the heightened sensitivity to developmental changes.

It is important to note that this study did not account for gestational age, which introduces potential limitations when interpreting results from the first 1–2 months of life. Inclusion of gestational age would allow for a more accurate characterisation of peripheral nervous system maturation from preterm through to full-term infants. While this omission likely increases variability in parameter estimates during early infancy, it is unlikely to have a significant impact on results beyond two months of age.

In the supplemental data, we provide clustering plots for each nerve. Visual inspection of these plots shows that the clusters derived using K-means clustering and Silhouette analysis contain a mix of concordant and discordant groupings. The discordant groupings highlight a potential limitation of the study; however, when examining each estimated parameter individually, it is sometimes challenging to visually distinguish which data points correspond to the normal and abnormal populations. A further issue with dividing the data into two groups is that, if the distributions of the nerve parameters substantially overlap, visually separating the groups becomes nearly impossible, and an automatic clustering approach can partially address this limitation. Moreover, when assessing age-related changes in nerve parameters, the final values were calculated across several age categories, as illustrated in Fig. 1, Fig. 2, Fig. 3. This procedure helps to minimise the influence of any misclassified clusters on the final estimates of nerve values.

The distributions of our normative data were compared with previously reported values. Notably, the literature shows substantial variation in both measured amplitudes and conduction velocities. Several factors likely contribute to this variability, including differences in study technique, electrode type, and hardware and software configurations (Kimura et al., 1988, Tankisi et al., 2020). Amplitude measurements exhibit the greatest variability, partly attributable to hardware differences that lie beyond the clinician’s control. This is particularly relevant in clinical settings where multiple nerve conduction systems are used, as low-amplitude signals such as SNAPs frequently vary markedly between machines and configurations (Koo et al., 2012). Our data shows relatively high SNAP amplitudes and low CMAP amplitudes, again due to a mixture of method and hardware. Conduction velocities also differ, influenced in particular by electrode placement—especially when short inter-electrode distances are used. Population characteristics also vary across reports: some studies present normative data from strictly healthy cohorts, whereas others include mixed populations containing individuals with various neuromuscular conditions. Compounding these factors is the difficulty of assembling sufficiently large cohorts, especially in very young age groups, and of reliably distinguishing datasets containing pathological values from those that do not. These issues collectively contribute to the wide spread of reported values. These observations underscore the importance of each laboratory establishing its own normative data—whether extrapolated computationally or derived from a carefully selected non-pathological cohort—to ensure consistency and clinical relevance within its specific technical environment. The values we present align with this broader body of published data and fall within the ranges reported in the literature (Vecchierini-Blineau and Guiheneuc, 1979, Kang, 2017, Garcia et al., 2000, Moglia et al., 1989, Jabre et al., 2020, Ryan et al., 2019, Shelly et al., 2023).

## 5 Conclusion

In this study, we analysed electroneurography data collected over a 15-year period at a tertiary paediatric hospital. Using an unsupervised clustering technique combined with exponential fitting and age as a covariate, we estimated normative values across a wide developmental range. Most changes occurred during the first years of life, underscoring the importance of dense sampling in early childhood.

This work demonstrates that unsupervised clustering can derive normative paediatric electroneurography values directly from clinical datasets. The approach is practical, generalisable, and capable of identifying likely healthy individuals using multivariate parameters. Furthermore, the methodology provides a standardised framework for estimating normative ranges, which can be applied in electrophysiological laboratories to generate site-specific reference values.

## CRediT authorship contribution statement

**G.K. Cooray:** Conception and design of the study, Data analysis, Statistical analysis, Preparing tables and figures, Writing and editing the text. **D. Motan:** Data extraction and pre-analysis, Writing and editing the text. **K. Howse:** Data extraction and pre-analysis, Writing and editing the text. **L. Nastasi:** Conception and design of the study, Writing and editing the text. **J. Deeb:** Conception and design of the study, Writing and editing the text.

## Ethical review

This study was reviewed by the local hospital review board and approved (ref number: 4071)

## Declaration of Generative AI and AI-assisted technologies in the writing process

Statement: During the preparation of this work the author(s) used ChatGPT 4 to nuance the language. After using this tool, the authors reviewed and edited the content as needed and take full responsibility for the content of the publication.

## Declaration of funding

No specific funding was received during the study.

## Declaration of competing interest

None of the authors have potential conflicts of interest to be disclosed.

## Appendix A Supplementary data

MMC S1 Supplementary data describe the nerve conduction protocol, derived clusters, and the approach used to assess normal proxies. MMC S1
