# Supplementary material for: Estimating paediatric normative values for nerve studies using clustering techniques
Source: Clin Neurophysiol Pract. 2026 Feb 28;11:187–94. doi: 10.1016/j.cnp.2026.02.006 (PMC12996837; doi:10.1016/j.cnp.2026.02.006)
Supplement: MMC S1 — Supplementary data describe the nerve conduction protocol, derived clusters, and the approach used to assess normal proxies. [file mmc1.pdf]

Supplementary Material: Motor and Sensory Clustering Results

G.K. Cooray, D. Motan, K. Howse, L. Nastasi, J. Deeb

February 12, 2026

1 Nerve conduction studies

Nerve conduction studies (NCS) were carried out using the Keypoint Net system (Dantec KeyPoint; Alpine Biomedical Aps, Skovlunde, Denmark). The standard amplifiers integrated in the system were used for both sensory and motor nerve recordings (band-pass filter settings were 20 Hz to 5 kHz for sensory studies and 20 Hz to 10 kHz for motor studies). Electrical stimulation was applied using felt-pad surface electrodes available in two sizes, suitable for infants and older children. The inter-electrode distance was 10 mm for the infant stimulator and 25 mm for the larger stimulator. Nerve responses were captured with disposable adhesive surface electrodes (mbu blue sensor ref NF-10-SC/12, Ambu A/S, Baltorpbakken 13, DK-2750 Ballerup). The ground electrode, identical in size and material to the recording electrodes, was positioned between the stimulation and recording sites. These electrodes have a rectangular recording surface with a circular adhesive border and were trimmed, when necessary, to accommodate very small limbs. Skin temperature was monitored and maintained at 32 °C, when possible.

1.1 Motor nerve studies

1.1.1 Median nerve

Compound muscle action potentials (CMAPs) were obtained from the abductor pollicis brevis (APB) muscle. The active (cathodal) recording electrode was positioned over the APB muscle belly, located at the midpoint of the thenar eminence. The reference (anodal) electrode was placed on the distal phalanx of the thumb. Distal stimulation was delivered at the wrist, between the tendons of the palmaris longus and flexor carpi radialis muscles, at the midpoint of the distal wrist crease (with adjustments made for hand size). Proximal stimulation was administered at the medial side of the elbow, medial to the biceps tendon and just above the elbow crease.

1.1.2 Ulnar nerve

CMAPs were obtained from the abductor digiti minimi (ADM) muscle. The active recording electrode was positioned over the muscle belly on the ulnar side of the hypothenar eminence, and the reference electrode was placed on the proximal phalanx of the fifth digit. Distal stimulation was delivered at the wrist, just lateral to the flexor carpi ulnaris tendon, at the level of the distal wrist crease. Proximal stimulation was administered below the elbow, distal to the medial epicondyle, with additional stimulation applied above the elbow along the medial surface of the arm while the elbow was flexed at 90 degrees, if necessary.

1.1.3 Peroneal (fibular) nerve

CMAPs were obtained from the extensor digitorum brevis (EDB) muscle. The active electrode was positioned on the muscle belly on the dorsum of the foot, lateral to the extensor hallucis longus tendon. The reference electrode was placed at the fifth metatarsophalangeal joint. Distal stimulation was delivered at the ankle, at the midpoint on the anterior aspect of the ankle joint, lateral to the tibialis anterior tendon. Proximal stimulation was administered at the fibular head, just distal and lateral to the fibular head, and, if necessary, in the superior lateral region of the popliteal fossa.

1.1.4 Tibial nerve

CMAPs were obtained from the abductor hallucis muscle. The active electrode was positioned over the muscle belly on the medial side of the foot, and the reference electrode was placed at the base of the great toe. Distal stimulation was delivered posterior to the medial malleolus, in the region between the malleolus and the Achilles tendon. Proximal stimulation was administered in the popliteal fossa, medial to the midline and just above the popliteal crease.

1.2 Sensory nerve studies

1.2.1 Median nerve

Sensory nerve action potentials (SNAPs) were obtained orthodromically from the mid-palm. Adhesive recording electrodes were positioned at the wrist, over the distal wrist crease, between the palmaris longus and flexor carpi radialis tendons. Stimulation was delivered at the mid-palm between the superficial flexor tendons to digits II and III. The stimulation-recording distance varied with the child's age and the available space for electrode placement on the palm, ranging from 8 cm in older children to as short as 3 cm in infants.

1.2.2 Ulnar nerve

SNAPs were obtained orthodromically from the lateral side of the wrist. Adhesive recording electrodes were positioned at the wrist, just lateral to the flexor

carpi ulnaris tendon at the distal wrist crease. Stimulation was delivered over the lateral aspect of the palm.

1.2.3 Medial plantar nerve

SNAPs were obtained orthodromically from the medial plantar region of the foot, using a recording electrode positioned along the medial side of the lower leg, posterior to the medial malleolus, with the reference electrode aligned with the active electrode but placed more proximally on the medial aspect of the lower leg. Stimulation was delivered on the medial side of the sole, with the stimulating electrode positioned between the flexor tendons of the hallux and the second toe.

1.2.4 Sural nerve

SNAPs were obtained using antidromic recording, positioning the active electrode posterior to the lateral malleolus or slightly more proximally, with the reference electrode placed distally. Stimulation was delivered over the posterolateral region of the calf.

1.2.5 Superficial peroneal nerve

SNAPs were obtained antidromically from the dorsum of the foot, positioned lateral to the extensor digitorum longus tendon. Electrical stimulation was delivered to the anterolateral region of the lower leg, at the junction between the middle and distal thirds of the leg.

1.3 Definition of estimated nerve conduction parameters

1.3.1 Compound muscle action potential (CMAP) parameters

CMAP amplitude was measured from the baseline to the peak of the initial negative deflection (negative peak amplitude) following distal stimulation and is reported in millivolts (mV). CMAP distal latency was defined as the time interval between the stimulus artefact and the onset of the initial negative deflection of the CMAP waveform, measured in milliseconds (ms). CMAP duration was measured from the onset of the initial negative deflection to the return to baseline of the final negative phase. CMAP area was calculated as the area under the negative phase of the waveform and expressed in millivolt–milliseconds (mV·ms).

1.3.2 Motor nerve conduction velocity (MCV)

Motor nerve conduction velocity was determined by dividing the distance between the proximal and distal stimulation points by the difference in CMAP onset latencies recorded at these two locations. Conduction velocity is expressed in metres per second (m/s). The distances between stimulation points were

obtained by measuring along the anatomical path of the nerve with a flexible measuring tape.

1.3.3 Sensory nerve action potential (SNAP) parameters

SNAP amplitude was determined as the voltage difference from the peak of the first negative deflection to the following positive peak (peak-to-peak amplitude) and is expressed in microvolts (µV). SNAP onset latency was defined as the time from the stimulus artefact to the beginning of the initial negative deflection and was measured in milliseconds (ms).

1.3.4 Sensory nerve conduction velocity (SCV)

Sensory nerve conduction velocity was determined by dividing the distance between the stimulation and recording sites by the onset latency of the SNAP. Conduction velocity is expressed in metres per second (m/s). All distances were taken along the anatomical pathway of the nerve.

2 Clustering Figures

Clustering of the mixed data was performed on datasets containing 50 data points, where each point comprised the relevant nerve study parameters. For the motor studies, each data point included four parameter values: CMAP amplitude, conduction velocity (CV), distal motor latency (DML), and negative CMAP area. For the sensory studies, each data point included two parameters: SNAP amplitude and CV.

The figures below show sample sets of 50 data points at ages 1, 3, 6, 12, and 16 years. The blue distribution represents the proxy for normative values and was selected as the cluster with the largest CMAP amplitude. Motor clusters are shown in Figures 1–4, and sensory clusters are shown in Figures 5–8.

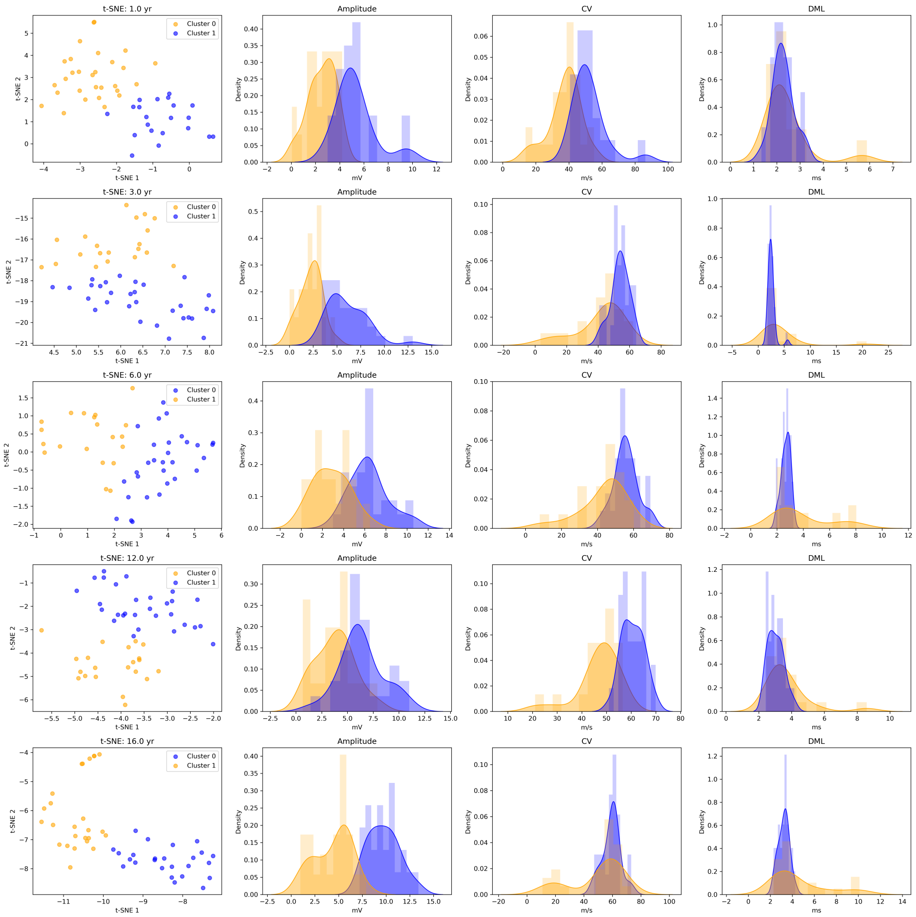

Figure 1: Clustering of data points for motor median. First column shows t-SNE scatter plots with two different clusters. Column 2-4 shows with CMAP, CV and DML for each of the clusters. The blue cluster is used as a proxy for normal data.

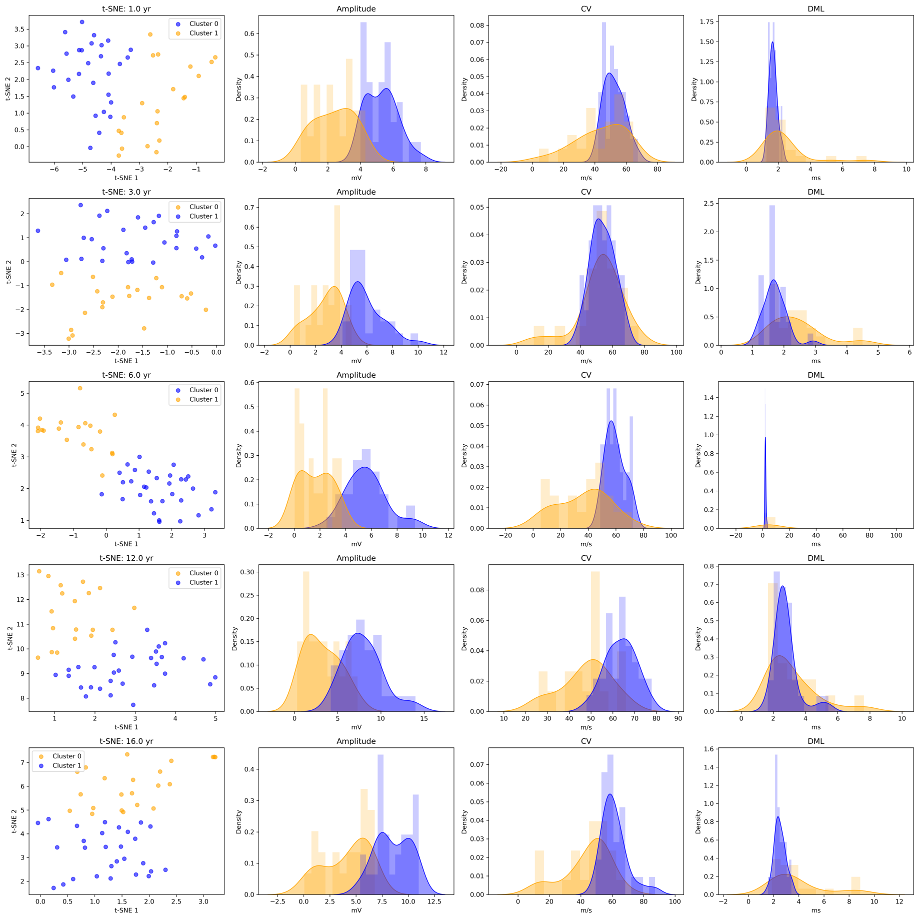

Figure 2: Clustering of data points for motor ulnar. First column shows t-SNE scatter plots with two different clusters. Column 2-4 shows with CMAP, CV and DML for each of the clusters. The blue cluster is used as a proxy for normal data.

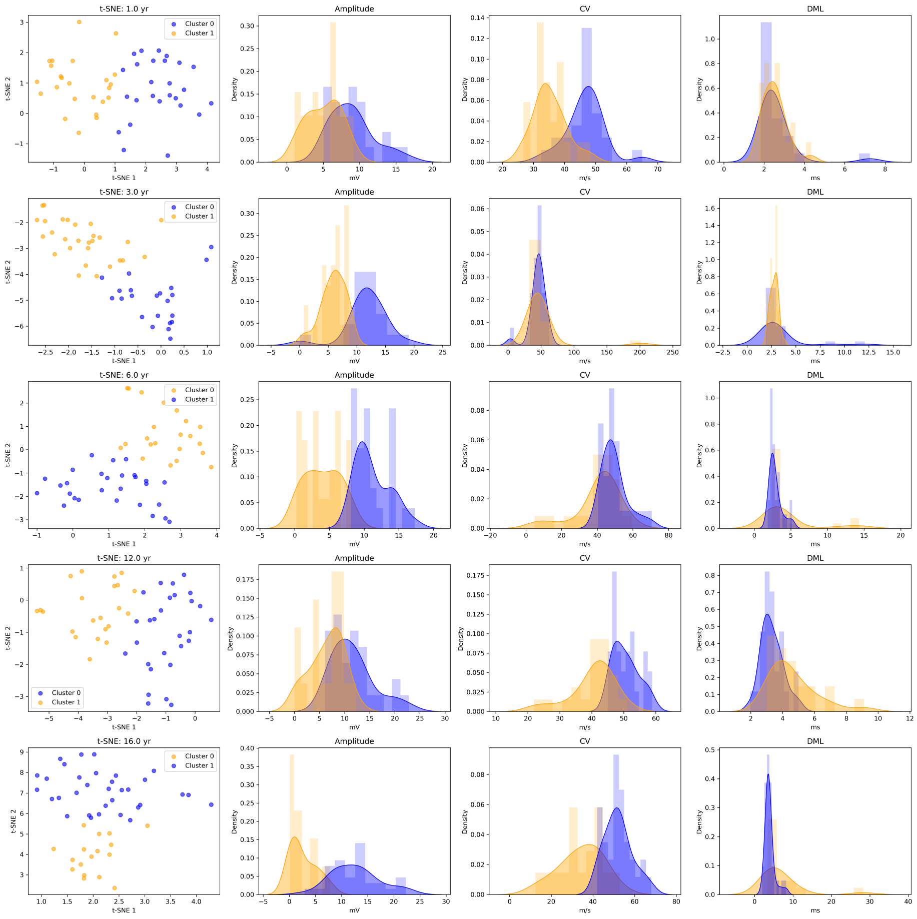

Figure 3: Clustering of data points for motor tibial. First column shows t-SNE scatter plots with two different clusters. Column 2-4 shows with CMAP, CV and DML for each of the clusters. The blue cluster is used as a proxy for normal data.

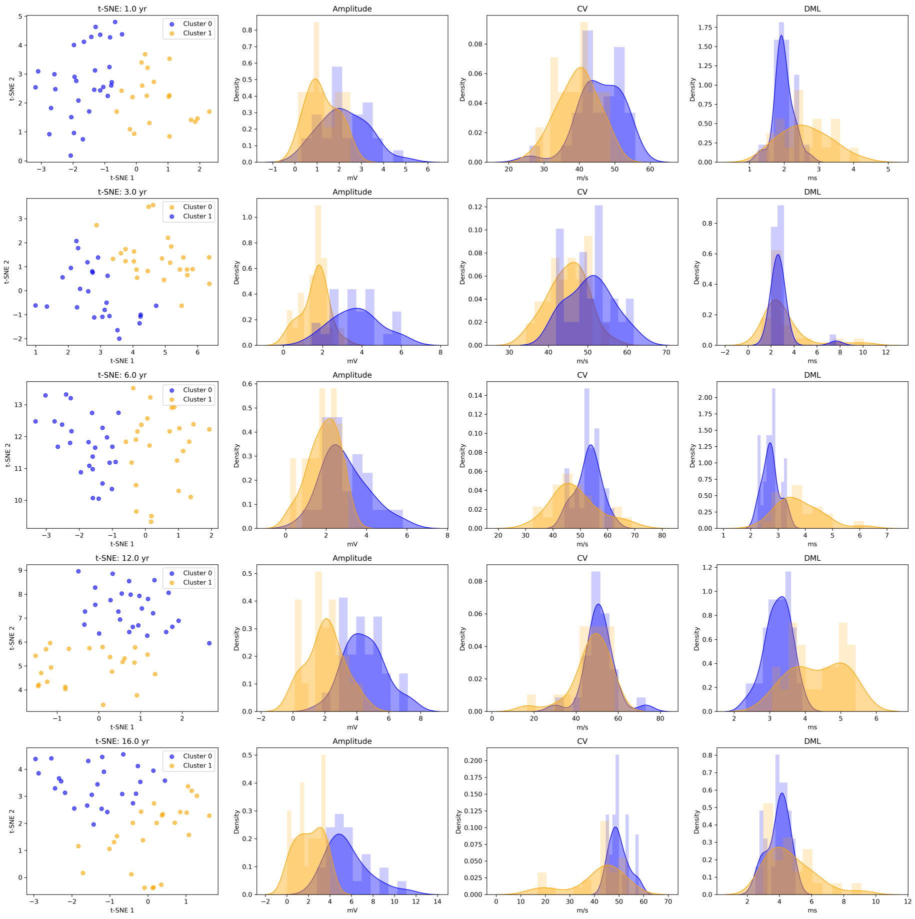

Figure 4: Clustering of data points for motor peroneus. First column shows t-SNE scatter plots with two different clusters. Column 2-4 shows with CMAP, CV and DML for each of the clusters. The blue cluster is used as a proxy for normal data.

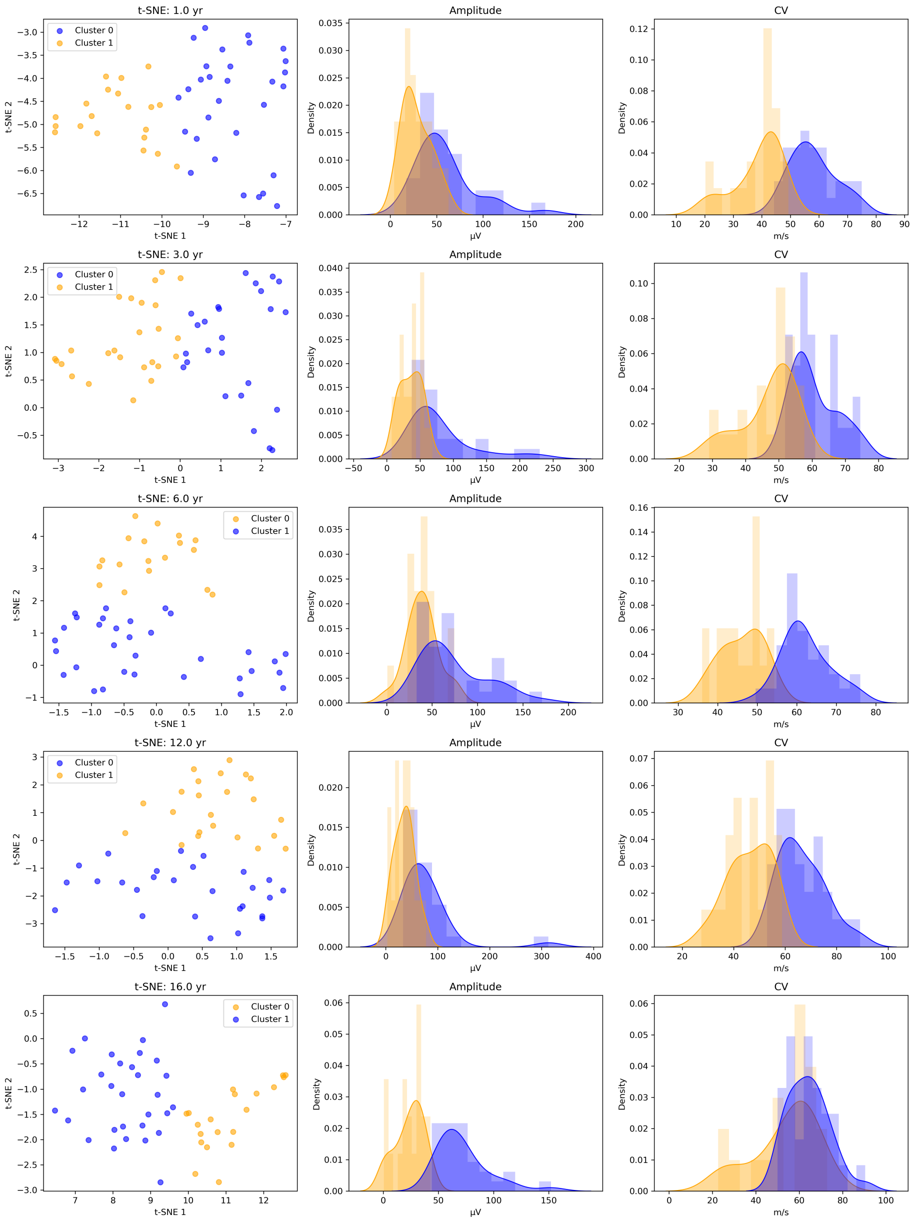

Figure 5: Clustering of data points for sensory median. First column shows t-SNE scatter plots with two different clusters. Column 2- shows with CMAP, CV and DML for each of the clusters. The blue cluster is used as a proxy for normal data.

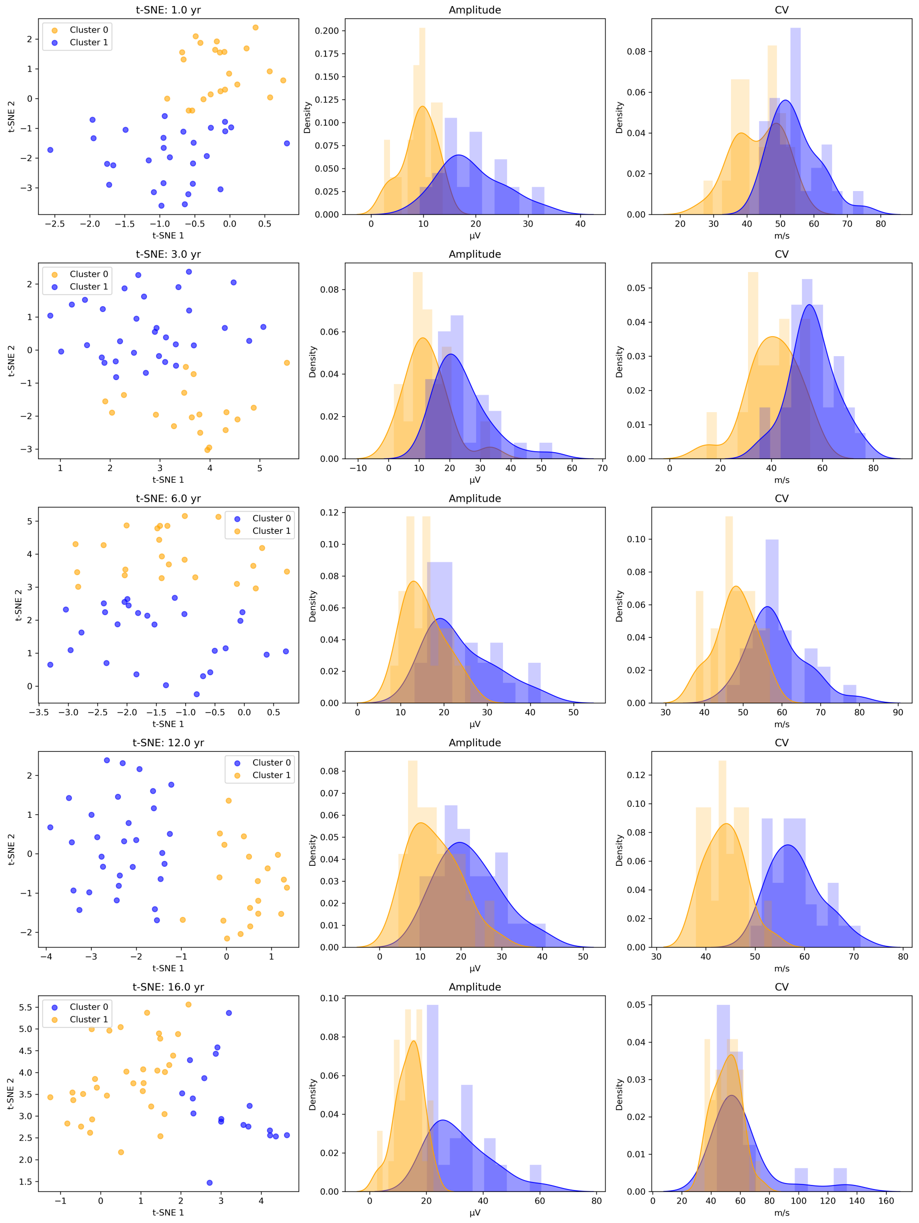

Figure 6: Clustering of data points for sensory sural. First column shows t-SNE scatter plots with two different clusters. Column 2-4 shows with CMAP, CV and DML for each of the clusters. The blue cluster is used as a proxy for normal data.

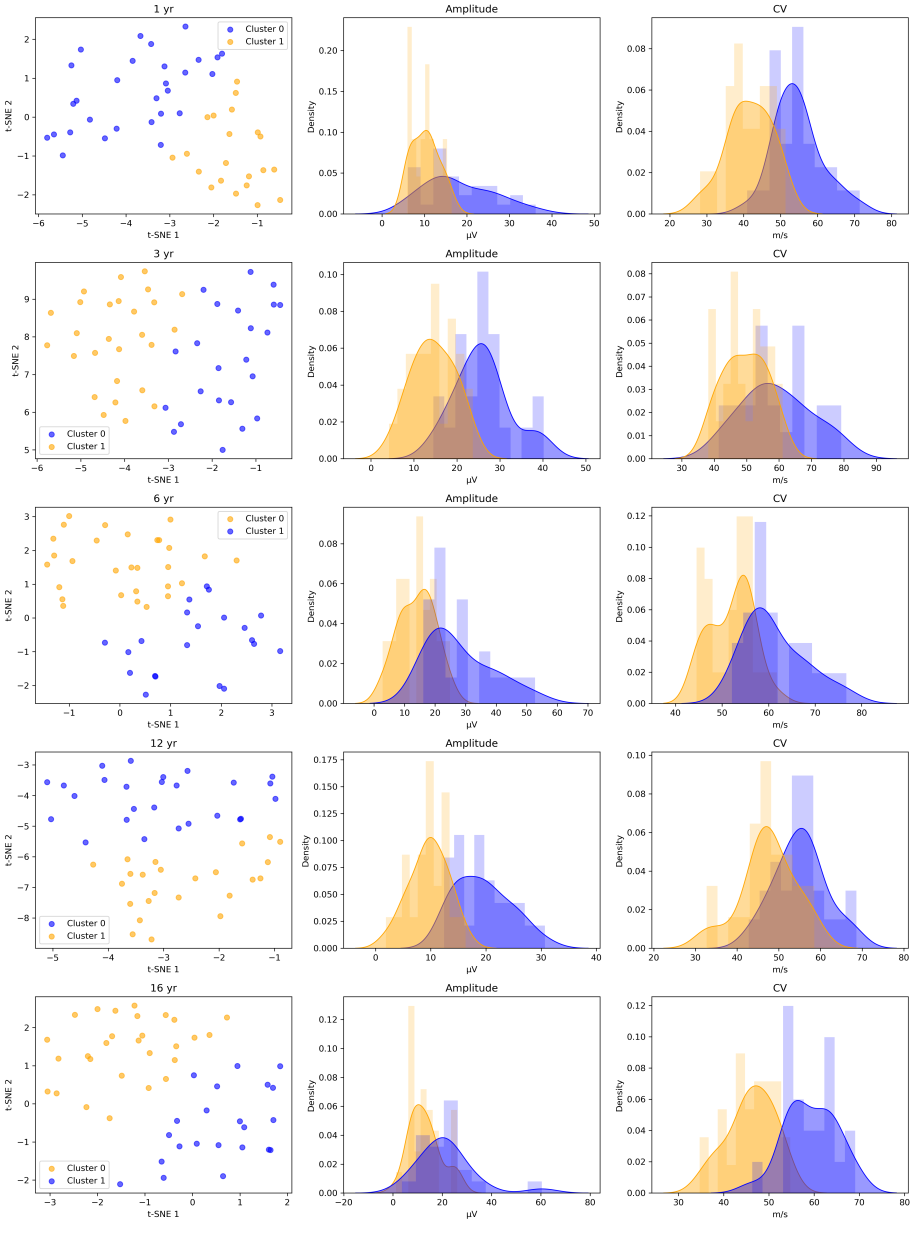

Figure 7: Clustering of data points for sensory peroneus (superficialis). First column shows t-SNE scatter plots with two different clusters. Column 2-4 shows with CMAP, CV and DML for each of the clusters. The blue cluster is used as a proxy for normal data.

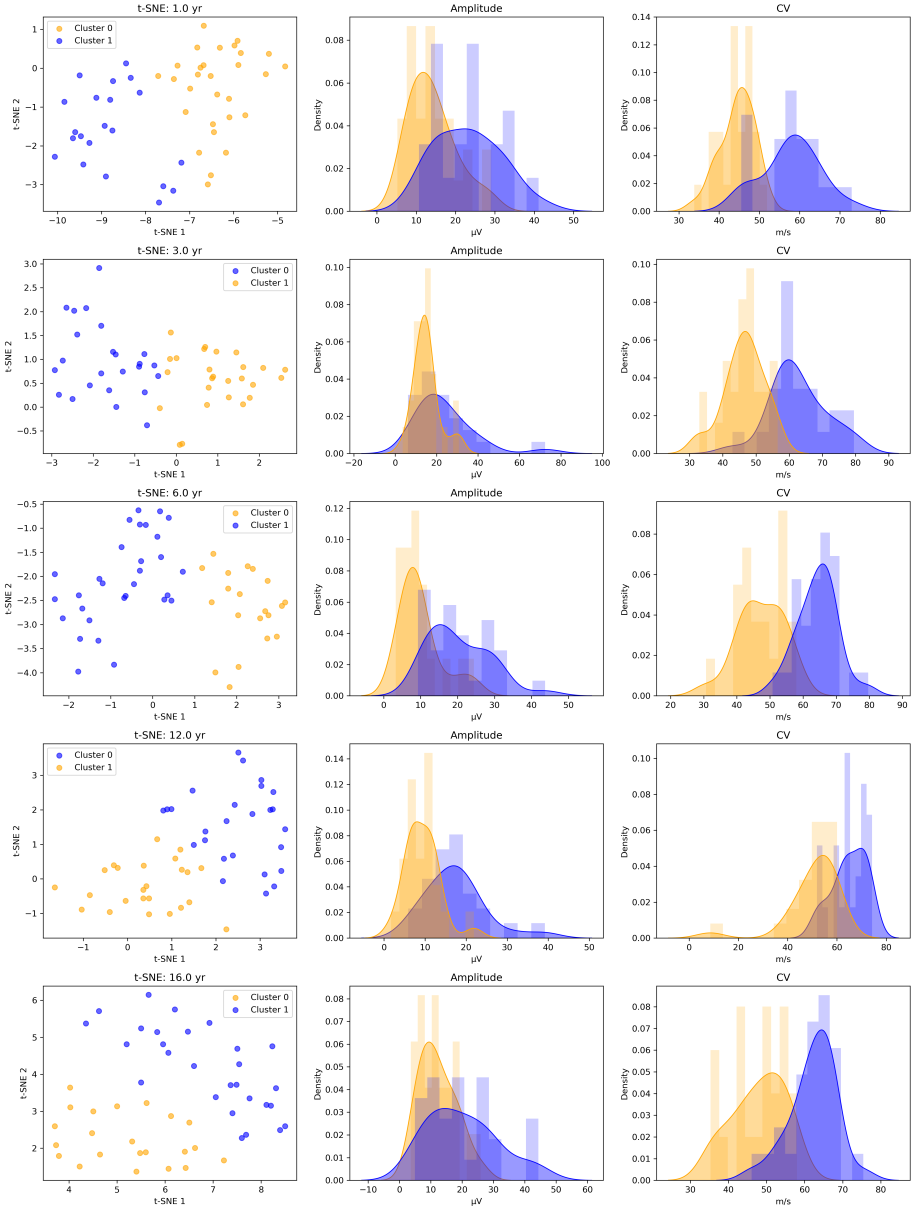

Figure 8: Clustering of data points for medial plantar nerve. First column shows t-SNE scatter plots with two different clusters. Column 2-4 shows with CMAP, CV and DML for each of the clusters. The blue cluster is used as a proxy for normal data.
